# Bacterial DNA Detection in the Blood of Healthy Subjects

**DOI:** 10.52547/ibj.26.3.230

**Published:** 2022-03-13

**Authors:** Javad Raeisi, Mana Oloomi, Mohammad Reza Zolfaghari, Seyed Davar Siadat, Mohsen Zargar, Zahra Pourramezan

**Affiliations:** 1 Department of Microbiology, Qom Branch, Islamic Azad University, Qom, Iran;; 2 Department of Molecular Biology, Pasteur Institute of Iran, Tehran, Iran;; 3 Department of Mycobacteriology, Pasteur Institute of Iran, Tehran, Iran

**Keywords:** Bacteria, Blood, Microbiome, Sequencing, 16S rRNA

## Abstract

**Background:**

The presence of microbiome in the blood samples of healthy individuals has been addressed. However, no information can be found on the healthy human blood microbiome of Iranian subjects. The current study is thus aimed to investigate the existence of bacteria or bacterial DNA in healthy individuals.

**Methods:**

Blood samples of healthy subjects were incubated in BHI broth at 37 °C for 72 h. The 16S rRNA PCR and sequencing were performed to analyze bacterial isolates. The 16S rRNA PCR was directly carried out on DNA samples extracted from the blood of healthy individuals. NGS was conducted on blood samples with culture-positive results.

**Results:**

Fifty blood samples were tested, and six samples were positive by culture as confirmed by Gram staining and microscopy. The obtained 16S rRNA sequences of cultured bacterial isolates revealed the presence of *Bacilli* and *Staphylococcus* species by clustering in the GeneBank database (≥97% identity). The 16S rRNA gene sequencing results of one non-cultured blood specimen showed the presence of *Burkholderia*. NGS results illustrated the presence of *Romboutsia*, *Lactobacillus*,* Streptococcus*,* Bacteroides, *and* Staphylococcus *in the blood samples of positive cultures.

**Conclusion:**

The dormant blood microbiome of healthy individuals may give the idea that the steady transfer of bacteria into the blood does not necessarily lead to sepsis. However, the origins and identities of blood-associated bacterial rDNA sequences need more evaluation in the healthy population.

## INTRODUCTION

The traditional belief of blood sterility in healthy humans has been challenged^[1]^. The first report on the presence of external cells in human blood dates back to 1969 when Tedeschi *et al.*^[2]^ announced the existence of L-phase or mycoplasma-like bacteria in the blood of healthy subjects. According to Gerald Domingue and Jorgen Schlegel (reviewed in^[3]^), 7% of filtered blood samples of healthy individuals exhibited bacterial growth. Several recent studies have confirmed the presence of bacteria in the blood of healthy subjects^[4-13]^. Various methods have been used for the investigation of the blood microbiome, including qPCR, and rRNA gene-specific fluorescent probes^[4]^, sequencing of PCR-amplified 16S rRNA^[5-7,9]^, and blood culture^[8,11]^. 16S targeted metagenomic sequencing (Illumina MiSeq)^[7,8,10]^ and 16S rRNA RT-qPCR^[9]^ were also employed. Despite the technical difficulties of bacterial blood cultivation, many research groups managed to culture and microscopically observe bacteria in healthy blood individuals^[6,12]^. The novel theory of healthy blood microbiota rises the possibility of the clinical sepsis incidence associated with blood transfusion^[7,13,14]^ with an prevalence of 2.5 per 100,000 human blood transfusions in France^[15]^ and one clinical sepsis in every 100,000 platelet transfusions reported in the United States^[16]^. Due to the potential severity and lethal threat of transfusion-transmitted bacterial infection through blood transfusion, our understanding of the blood microbiome in healthy individuals can result in the development of a sensitive and exhaustive screening method to detect a healthy condition in blood donors^[7]^. 

 The origin of blood microbiome remains undefined; however, several data have suggested a translocation into the circulatory system from different body sites, such as the gastrointestinal tract, skin, oral cavity, or translocation driven by cells of the immune system. It has been observed that interindividual variations in the extent of the blood microbiome are significantly associated with geographical origin rather than the age of the subject analyzed. This observation, together with the genetic peculiarities of each geographical area, could reflect cultural/behavioral features such as diet, environmental exposure, climate, and social habits^[17]^. There is a hypothesis that the origin of bacteria in the blood is a consequence of gastrointestinal microbial translocation^[18]^. In addition, dysbiosis in the blood-borne bacterial microbiome may stimulate pathogenesis. Therefore, the blood microbiome can consider as a potential biomarker of human health^[17].^

In this study, the blood microbiome of healthy individuals was investigated by culture, 16S rRNA sequencing, and NGS of culture positive blood samples. The current study is thus aimed to investigate the existence of either bacteria or bacterial DNA in healthy individuals. Different types of microbiome profiles have been reported in varied countries, even different regions in a country. To the best of our knowledge, there are no reports on the blood microbiome of the Iranian population. The microbiome profile may differ from the normal population depending on the environment and culture. Normal microbiome could be changed based on various diseases as investigated in this study.

## MATERIALS AND METHODS

**Blood samples**

Blood samples were collected from 50 healthy adults at the Pasteur Institute of Iran, Tehran from January to November 2019. The age of volunteers was between 25 and 60 years old, and they did not suffer from infectious diseases. The exclusion criteria included any cancer types, autoimmune (thyroid disorders, multiple sclerosis, and type 1 diabetes) and gastrointestinal (Crohn's disease and irritable bowel syndrome) diseases, any contagious infection disease, dermatitis, cardiovascular diseases, and metabolic syndrome. Volunteers under specific medical treatments such as antibiotics were also excluded. The included subjects filled a questioner and signed a consent letter. The 50 healthy blood donors encompassed 30 women and 20 men in the age range of 25-53.

**Blood culture**

The blood sample (5 ml) from each donor was immediately cultured in an enrichment BHI medium using a sterile syringe under a Class II biologic safety cabinet. The inoculated medium was then incubated at 37 °C for 72 h^[7]^. Subculturing of trypticase soy agar and blood agar media were provided to further purify bacterial cells. Identification of isolated colonies was based on standard biochemical techniques, including Gram staining and microscopy, catalase, and sugar fermentation (triple sugar iron agar). Polymixin B (300 UIE; Biomeriex, USA) and novibiocin (30 MCG; Difco, France) were also used for further characterization. Negative controls were considered in all practical steps. The selected samples were cultured in BHI (Merck, USA). After overnight incubation, samples were centrifuged and mixed with 30% glycerol and kept at -80 °C. Then 16S rRNA gene sequencing was carried out on a pure culture for the identification of the isolates.

**DNA extraction**

DNA extraction was carried out from the whole blood (200 μL) of the isolated strains (1 mL of overnight culture) using a Class II biologic safety cabinet through a PCR template preparation kit as instructed by the manufacturer (Roche, Germany). The DNA extraction was designed to minimize the risk of sample contamination. The quantity and quality of extracted DNA were assessed by 1% agarose gel electrophoresis (in Tris-Borate-EDTA) and by a microplate reader (BioTek, USA). All DNA extracts were kept at -20 °C until further processing.

**PCR amplification**

The 16S rDNA region was amplified by PCR (V3-V4) in a thermal cycler (Eppendorf, Germany). The PCR thermal cycles were as follows: 95 °C for 3 min, followed by 30 cycles at 95 °C for 30 s, 55 °C for 30 s, and 72 °C for 45 s and a final extension at 72 °C for 10 min. The primer set 27F (5’-TTGGAGAGTTT GATCCTGGCTC-3’) and 1492R (5’-AGGAGGTGAT CCAACCGCA-3’)^[19]^ as universal primers, was used to amplify extracted rRNA gene fragment. This universal PCR can amplify the relevant fragments from all bacteria that have been examined to date^[20]^. The PCR reactions were carried out in a 20-μL mixture in triplicate containing 2 μL of 10× PCR buffer, 2 μL of 2.5 mM of dNTPs, 0.8 μL of each primer (5 μM), 0.2 μL of Taq DNA polymerase (TaKaRa Bio, Dalian, China), and 10 ng of template DNA^[21-23]^. Also, the corresponding universal *primer* sets were used as library pools for sequencing on the Illumina MiSeq (NGS). The bacterial species with 99% or higher similarity and the genus with 90-99% were considered, while the score <90% was interpreted as an unidentified organism. 

**N** **ext generation sequencing**

Taxonomic diversity in blood samples can be explored by amplifying hyper-variable regions (V3 and V4) of 16S rRNA. The following universal external primers were applied for the amplification of the conservative V3 and V4 regions of 16S rRNA gene: 341F (CCTAYGGGRBGCASCAG), 805R (GGACTA CNNGGGTATCTAAT)^[^^16]^, 515F (GTGCCAGCMGC CGCGGTAA), and 806R (GGACTACHVHHHTW TCTAAT)^[21]^. DNA amplification was performed using PCR Master Mix Phusion® (New England Biolabs, USA) as previously described^[22]^. Sequencing libraries were established by NEBNext ®, Ultra DNA Library PreKit for Illumina according to the manufacturer’s guidelines and added index codes **(***New England Biolabs*, *China)*. The quality of the library was evaluated by the Qubit@ 2.0 Fluorometer and Agilent Bioanalyzer 2100 System (Thermo Scientific, USA). The sequencing procedure was performed on the Illumina platforms at Novogene Bioinformatics Technology Co., Beijing, China.

**Sequence analysis**

The original DNA fragments were merged by FLASH (V1.2.7, http://ccb.jhu.edu/software/FLASH/) and pairing the reads^[21].^ The tags were compared with the reference databases (Gold database; http://drive5. com/uchime/uchime_download.html) to detect and remove the chimera sequences^[22]^. Sequence analyses were achieved by Uparse software (version 1.0.1001^[23,24]^. Sequences with ≥97% similarity were allocated to the same OTU. 

**Data analysis and statistics**

A cross-sample comparison of OTUs frequency was carried out by non-parametric ANOVA (Kruskal-Wallis test). The difference in the abundance of OTUs between the groups was assessed by DESeq2 (negative binomial Wald test). 

## RESULTS

**Fig. 1:**
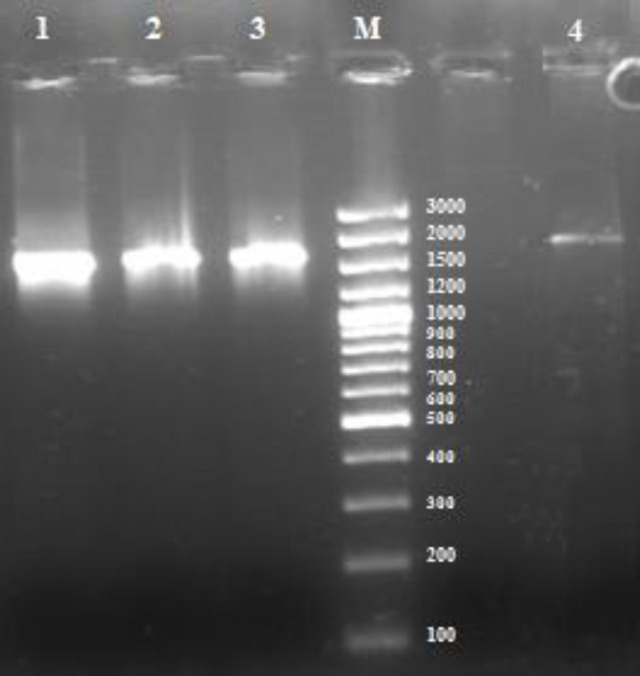
Agarose gel electrophoresis and ethidium bromide staining of 16s rRNA PCR product proliferated from (lane 1) *E. coli* k012 genome (positive control), (lanes 2 and 3) isolates of bacterial strains from blood culture, and (lane 4) whole blood PCR-positive sample 7, and (M) 1 kb ladder.

**Fig. 2:**
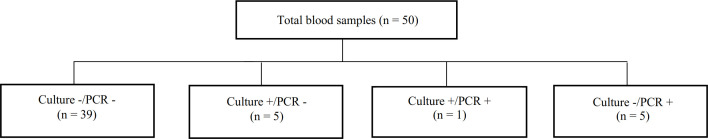
PCR test of 16S rRNA gene versus culture technique for the detection of the blood microbiome.

Fifty samples were collected from healthy individuals with no history of inflammatory disease or bacteremia. Bacterial growth was detected in six culture-positive blood samples under aerobic conditions. All of the culture-positive samples were cultured anaerobically. Six positive samples were detected for total blood DNA template (six females) using 16S rDNA PCR, while six positive samples were detected by culture method (one male and five females), as represented in Figure 1. Based on both techniques, one of the samples was positive, and 39 samples were negative. Figure 2 compares both culture and PCR methods. 

**Fig. 3:**
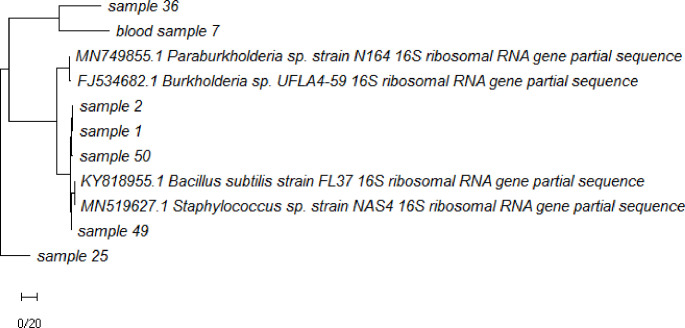
Phylogenetic tree based on 16S rRNA nucleotide sequences of the PCR product of isolated strains from blood cultures and one blood genome PCR positive (sample 7) using the MEGA v7.0.14 program by UPGMA.

**Fig. 4:**
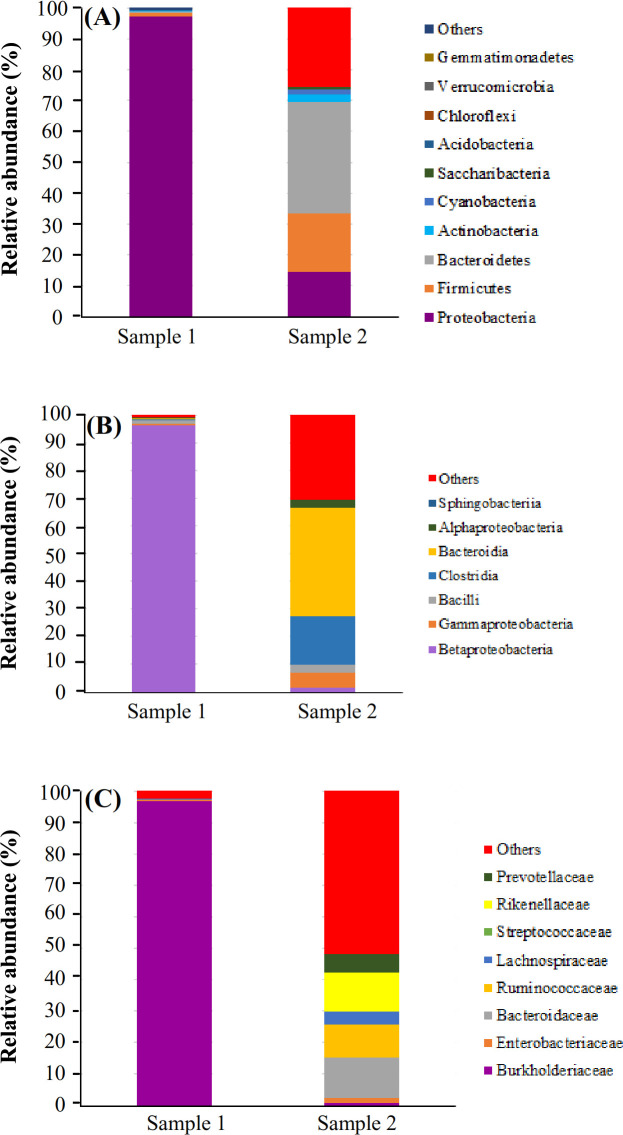
Relative abundance of OTU reads and composition of the putative blood microbiota of healthy women at the (A) phylum, (B) class, and (C) family levels assessed by 16S targeted metagenomic sequencing.

**Fig. 5:**
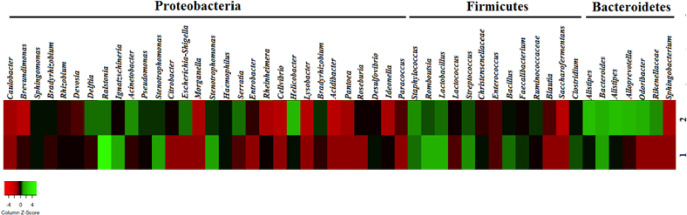
Heatmap showing the abundance of predominant blood microbiota of two healthy samples at the genus level, identified by taxonomic classification. The data represent the Log10 values of the OTU counts each genus.

According to the 16S rRNA sequencing, *Bacilli* and *Staphylococcus* genera were isolated from the cultures. These two strains showed similarity to *Staphylococcus epidermidis *(97.9% and 89.87%, respectively). One strain was similar to *S. hominis (*98.06%) and one to *Bacilli bacterium *(98.52%), one of them was similar to *S. epidermidis (*91.03 %) and to some extent to *Bacillus subtilis *(90.92%)*. *The phylogenetic tree built on the 1200 bp of the nucleotide sequence (16S rRNA PCR product) of strains isolated from blood cultures, and one sequence was obtained via direct PCR on blood genome (sample 7), as depicted in Figure 3. According to the NGS results, proteobacteria, bacteroidetes, and firmicutes were over-represented in these two healthy subjects. At the class level, betaproteobacteria, gammaproteobacteria, bacilli, clostridia, and alphaproteobacteria were observed (Fig. 4). Taxonomic differences at the genus level were relatively high among healthy individuals. Predominant blood-derived bacterial genera (Fig. 5) included *Romboutsia*, *Lactobacillus*,* Streptococcus*,* Bacteroides*, and* Staphylococcus*, which dominated the blood microbiota in these two positive blood samples. 

## DISCUSSION

The assessment of the blood microbiome in a normal population by NGS was the aim of this research to reach the typical microbiome in the population. Changes in the microbiome population may lead to different diseases. Therefore, this study contributes to the characterization of microbial normal population in Tehran. Numerous papers have reported strategies for detecting prokaryotic DNA in the blood^[3,6]^. Different methods have been developed to assess healthy blood microbiome within the last 20 years, as listed in Table 1. Damgaard *et al.*^[6] ^incubated blood aerobically and anaerobically on blue lactose plates and trypticase soy blood agar for seven days. Kroumova *et al.*^[25]^ and Torres *et al.*^[26]^ stated that enrichment of blood culture in a highly nutritious liquid medium (BHI broth) could shorten the cultivation duration^[27]^. In addition to the technical difficulties of blood culture, avoiding contamination and manipulation of the samples might cause an unreal microbiome^[28,29]^. In this study, six out of 50 blood samples (12%) were positive by the culturing method. The same results were reported by Damgaard *et al.*^[6]^ and Jiménez *et al.*^[30]^. Panaiotov *et al.*^[18]^ investigated blood samples from 28 healthy individuals and illustrated that all tested blood samples were culture positive, as confirmed by transmission electron microscopy and Gram staining. They declared that the optimal growth temperature was at 43 °C, and the blood microbiota was suppressed at 37 °C. They noticed morphological forms of Gram-stain in blood, which had earlier been described by Domingue and Woody^[28]^ as dense bodies. 

Our studies indicated Gram-positive stained microbial core structures surrounded by the Gram-negative coat. There is increasing evidence that bacteria may be present in “dormant” forms, a situation in which they cannot be simply cultured using the conventional methods^[12]^. Different molecular methods have been used for the detection of dormant blood microbiome, including qPCR^[29]^, PCR-amplified 16S rRNA^[3,4]^, 16S rRNA RT-qPCR^[5,9]^, 16S rRNA gene colony PCR^[8]^, and 16S targeted metagenomic sequencing (Illumina MiSeq)^[7,8,10]^. Despite the high sensitivity, PCR could result in false positives and negatives, which have to be confirmed with other methods. In this regard, alternative methods have been developed. The real positive ones were assessed by the powerful and new technique of NGS^[30-32]^. The 16S rRNA gene colony PCR and sequencing were used to identify the isolated strains in the current study. It has been shown that some isolates carry sequences with similarities below the threshold (typically 97%). Two of our positive strains were identified at the genus level rather than the species level. According to the NGS results of the current study, *Proteobacteria*,* Bacteroidetes*, and *Firmicutes* were overrepresented in these two healthy subjects. Based on 16S rRNA gene-targeted sequencing of V3-V, *Panaiotov et al.*^[18]^ have stated that *Proteobacteria *is the predominant phylum based on 16SRNA gene-targeted sequencing V3-V4, followed by *Actinobacteria*,* Planctomycetes*, and *Firmicutes *among non-cultivable samples and *Proteobacteria, Firmicutes*,* Actinobacteria*,* Bacteroidetes*,* Fusobacteria*, and *Cyanobacteria*. In the present study, *Firmicutes* (*Staphylococcus*) had the highest total relative abundance in the 16S sequencing in cultivable samples, which coincided with the results of Whittle *et al.*^[11]^ who declared that the identification of the bacteria via microbial culture experiments may be originated from the skin contaminants during the venipuncture. Furthermore, some other studies have proposed that the bacterial presence in the blood can be the consequence of bacterial diffusion into the blood from other tissues such as skin, nasal, and vaginal or oral mucosa such as the gastrointestinal tract^[8,33-39]^. Whittle and colleagues^[11]^ confirmed the presence of these bacteria in the dormant form (i.e. not contaminants) in the blood, which may be revived following the pregrowth in BHI broth^[11]^. Based on a recent study, microorganisms found in human blood may correspond to free bacteria in the dormant state^[12]^, or bacteria inside white and red blood cells, or free bacterial DNA due to immune degradation^[3,6,30,40]^. Bacteria such as *S. aureus* can survive in white blood cells^[41,42]^. Moreover, Grice *et al.*^[43]^ have reported the common presence of *S. aureus* in healthy blood samples, as confirmed in the current study. With the help of a culture-based approach, Damgaard *et al.*^[6]^ illustrated bacterial growth in the blood of 37 out of 60 healthy subjects. *Propionibacterium* *acnes* and *Bacilli*, *S. epidermis*, and *Micrococcus* were the dominant taxa detected in their study. In the current study, bacterial growth was detected in the blood of 10% of healthy subjects. *Staphylococcus *and *Bacillus* species were also detected, as confirmed by the NGS findings. The 16S rRNA gene of one noncultivable blood sample belonging to *Proteobacteria *(*Bulkhulderia* *sp*.) was also sequenced.

**Table 1:** Comparison of healthy blood microbome studies during last 20 years

**Ref.**	**Methods**	**Samples**	Results	**Conclusion**
3	Dark-field microscopy, fluorescent in situ hybridization (FISH) flow cytometry 16S rRNA and *gyrB *genes detection	Healthy (n= 25)	Pleomorphic bacteria (TEM) Culture positive (0%) 16S rRNA gene showed identity with *Stenotrophomonas* *Maltophilia * *Pseudomonas*	Pleomorphic viable bacteria in blood could not be cultivated in vitro by regular techniques.
				
4	16S ribosomal RNA genes (rDNA) PCR	Healthy (n= 2)	Positive bands* (*100%)* Aquabacterium*, *Stenotrophomonas*, *Budvicia*, *Serratia*, *Bacillus *and *Flavobacteria * No clone detected within the bacteroides-clostridium-lactobacillus cluster, which is indigenous to gastrointestinal flora.	Physical and immunological Barriers may destroyed Bacteria, and the released rDNA may be transfered into the bloodstream directly or carried by phagocytes.
				
6	Colony PCR targeting 16S rDNA	Healthy (n = 60)	Culture positive (35%) 21 of 60 RBC-fractions and (53%) 32 of 60 plasma-fractions Propionibacterium acnes (23%) Staphylococcus epidermidis (38%)	Viable bacteria are present in blood from healthy donors.
				
8	(V3 and V4) of 16S rRNA library preparation NGS sequencing	Healthy (n = 23) Sepsis (n= 62)	Healthy microbiome included: Bifidobacteriales (73.0%), Actinobacteria	bacteria might steadily transfer into the blood, but not necessarily cause sepsis; DNAemia
				
9	16S rRNA gene qPCR	Healthy (n = 9) Liver patients (n = 9)	19 of 53 bacterial species detected (36%) Healthy microbiome: Bacteroidetes (Bacteroidales), Firmicutes (Clostridiales).	They identified bacterial DNA in ~ 90% of the individuals’ blood without clinical evidences.
				
10	Blood culturing NGS sequencing	Healthy (n = 28)	Culture positive (100%) Proteobacteria (46%), Firmicutes (25%), Actinobacteria (14%), Bacteroidetes (6%), Fusobacteria (3%) and Cyanobacteria (2%)	The dormant blood microbiome is innate of the healthy individuals.
				
11	Blood culture 16S rRNA gene PCR sequencing	Healthy (n = 5) asthmatic (n = 5)	Bacterial cultures positive (80%) Bacterial isolates; *Staphylococcus, Micrococcus, Kocuria, Corynebacterium* and *Propionibacterium*.	Blood microbiome were possibly have originated from the oral or skin communities.
				
30	Real-time PCR 16S ribosomal DNA (rDNA**)**	Healthy (n = 4)	Large amount of rDNA in blood specimens from healthy individuals *Pseudomonas fluorescens *as possible contaminants of *Taq *(and AmpliTaq) polymerases *Propionibacterium* *Acnes* *Microbacterium schleiferi*	They cannot conclude whether the origins of this DNA are the skin or blood, or both.
				
47	Unmapped RNA sequencing	Healthy (n = 49) Three brain-related disorders (n = 143)	Proteobacteria, Firmicutes, and Cyanobacteria	
				
48	V3 region of the 16S rRNA gene was amplified	Healthy (n = 12) Severe acute pancreatitis (n = 50)	Actinobacteria (17%) Bacteroides (10%) Firmicutes (11%) Proteobacteria (61%)	They distinguished blood microbiomes of severe acute pancreatitis (SAP) patients from healthy controls.
				
49	RT-qPCR analyzing the presence of gut bacteria in blood samples	Healthy (n = 50) Type 2 diabetes patients (n = 50)	Gut bacteria were identified in blood at a higher rate in diabetic patients than in control subjects, and most of these bacteria were Gram-positive: *Streptococcus*, *C. coccoides *group	The high rate of gut bacteria in the circulation prescribed translocation of bacteria from the gut to the bloodstream.
				
Current study	Blood culture 16S rRNA gene PCR Pyro-sequencing	Healthy subjects (n = 50)	Bacterial cultures positive (12%) PCR positive (12%) Bacterial isolates: *Staphylococcus, Bacilli* Direct blood PCR-sequencing: Bulkholderia	Blood microbiome were possibly have derived from the skin and gut communities.

The link between the human microbiome and disorders, including obesity, inflammatory bowel disease, arthritis, and autism, is rapidly expanding. However, each species in the *microbiome* is genetically heterogeneous, comprising *individual *cells whose genomes are *different*. There are reports that the blood samples were predominated by the genus *Achromobacter*. To a lesser extent, the blood samples also comprise members of the *Pseudomonas*. In other reports, at the genus level, the genera *Paenibacillus*,* Escherichia*,* Acinetobacter*^[7]^, *Pseudomonas*^[5-7]^, and *Propionibacterium*^[5,6]^ were predominated. Bacteria isolated from the aerobic cultures included the following genera: *Staphylococcus*,* Micrococcus*,* Kocuria*,* Corynebacterium*, and *Propionibacterium*. Bacteria isolated from the anaerobic cultures were less variable and included members from the facultative anaerobic *Staphylococcus* genus belonging to the phyla *Actinobacteria* *(Corynebacterium*,* Kocuria*, and *Micrococcus*) and *Firmicutes* (*Staphylococcus*), which all were represented in 16S rDNA level data. The skin microbiome is dominated by members of the genera *Corynebacterium*,* Micrococcus*,* Staphylococcus*, and* Propionibacterium*^[11]^. Although there is a differential composition of the blood microbiome, the influence on the composition of the diet, age, seasonal variation, and host immunomodulation, environmental impacts, and exposure to new microbes are essential. However, the blood bacterial diversity varies in different studies. In 2008, Moriyama *et al.*^[4]^ identified a set of bacterial taxa in their study on bacteria from the blood of two healthy individuals comprising mostly *Bacillus*,* Flavobacteria*,* Stenotrophomas*, and *Serratia*. 

In our results, the presence of *Bacilli* and *Staphylococcus* species was obtained by 16S rRNA sequences of cultivable bacterial isolates. The 16S rRNA gene sequencing of one non-cultivable blood specimen showed the presence of *Burkholderia*. Results from NGS illustrated the presence of *Romboutsia*, *Lactobacillus*,* Streptococcus*,* Bacteroides*, and* Staphylococcus *in the blood samples of positive cultures that can originate from skin. As described by several researchers^[43-45]^, the majority of blood microbiome cannot be cultured with the available techniques due to technical restrictions^[46]^ and biological characteristics of bacteria (i.e. bacterial L-forms and dormant forms)^[41]^. Many bacteria are usually detected within human blood in a dormant state^[12]^. Therefore, culture-based methods cannot reliably approve the presence of the blood microbiome. Moreover, the failure of the PCR in five (10%) culture positive blood samples could be assigned to the presence of inhibitors in our DNA preparations from the blood. Other sensitive analytical techniques, such as targeted NGS and qPCR, support current evidence on the bacterial presence in healthy blood samples^[7]^. As confirmed by NGS, these findings require further investigation in a large population. Furthermore, varied microbial patterns of the studied individuals could be attributed to the differences in their environments. The origin of genetic diversity can be recognized in the human normal microbiome as a prominent modifier of disease, influencing the metabolisms and modulating interactions with drugs. Numerous probiotics or beneficial bacteria can be used for preventing or treating certain diseases. Some beneficial bacteria, such as *Faecalibacterium prausnitzii*, have been introduced for the treatment of irritable bowel syndrome. Moreover, disease prevention and treatment by targeting the microbiome have been explored. Treatment strategies on the basis of the microbiome diagnosis could be employed in future personalized medicine. However, the first step is the recognition of the human microbiota, which requires further investigations.

## DECLARATIONS

### Ethical statement

The above-mentioned sampling protocols were approved by the Ethical, QOM, Iran (ethical code: IR.IAU.QOM.REC.1398.019). Each participant signed an informed consent form.

### Data availability

The raw data supporting the conclusions of this article are available from the authors upon reasonable request. 

### Author contributions

JR, writing original draft; MO, conceptualization, supervision and funding acquisition; MRZ, supervision; SDS and MZ, project administration; ZP, data and statistical analysis and writing the manuscript. All authors have read and approved the final manuscript.

### Conflict of interest

None declared.

### Funding/support

Funding was supported by the Pasteur Institute of Iran, Grant No. 1046.
